# Targeting circulating tumor cell‒neutrophil interactions: nanoengineered strategies for inhibiting cancer metastasis

**DOI:** 10.1186/s12951-025-03522-8

**Published:** 2025-06-17

**Authors:** Yong Su, Mingjing Leng, Qingqing Yang, Wenbi Jiang, Gang Xiang, Ling Long, Xing Zhou

**Affiliations:** 1 School of Pharmacy and Bioengineering, Chongqing University of Technology, Chongqing, 400054 People’s Republic of China; 2 Yunnan Key Laboratory of Stem Cell and Regenerative Medicine, Rehabilitation School, Kunming Medical University, Kunming, 650500 People’s Republic of China; 3 School of Pharmaceutical Sciences and Yunnan Provincial Key Laboratory of Pharmacology for Natural Products, Kunming Medical University, Kunming, 650500 People’s Republic of China; 4 Department of Oncology, Xinqiao Hospital, Army Medical University, Chongqing, 400054 People’s Republic of China

**Keywords:** Tumor metastasis, Drug delivery systems, Nanomedicine, Circulating tumor cells, CTC‒neutrophil interaction

## Abstract

Metastasis remains the leading cause of cancer-related mortality, with a persistently poor prognosis for metastatic cancer patients despite extensive therapeutic efforts. Circulating tumor cells (CTCs), which detach from primary tumors and enter the bloodstream, can establish distant metastatic sites. These CTCs often form heterotypic clusters with white blood cells, especially neutrophils, through various interaction mechanisms, including intercellular adhesion, cytokine secretion, protease release, and the formation of neutrophil extracellular traps (NETs). These interactions enhance CTCs survival, proliferation, invasion, and transendothelial migration while simultaneously remodeling premetastatic niches and the tumor microenvironment. Consequently, pharmacologically disrupting CTC‒neutrophil crosstalk represents a promising strategy to curb metastatic spread and improve clinical outcomes. Recent breakthroughs in nanotechnology-based drug delivery systems have shown considerable potential in antimetastatic therapies, offering significant advantages over conventional treatments, which are often associated with severe side effects and limited efficacy. This review systematically explores nanoengineered strategies targeting CTC‒neutrophil interactions, addresses the current limitations and outlines future directions for developing clinically translatable nanotherapeutics.

**Graphical Abstract:**

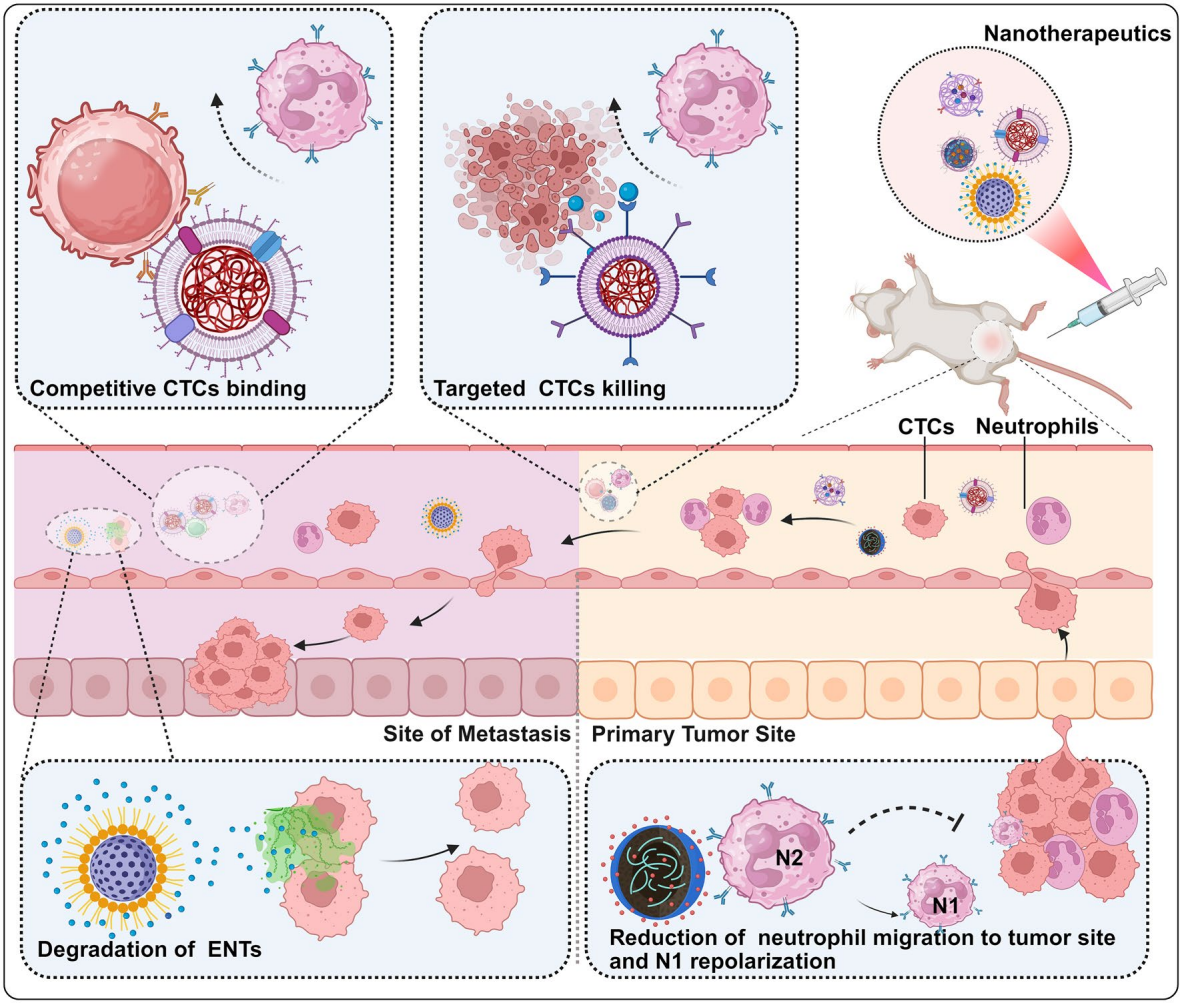

## Introduction

Metastasis remains the leading cause of cancer-related mortality [1] and involves a cascade of complex biological events, such as local invasion, extravasation, intravasation, micrometastasis formation, and proliferation within the primary tumor, premetastatic niches, vasculature, and metastatic sites [2]. For decades, systemic therapies—including chemotherapy, radiotherapy, immunotherapy, and molecularly targeted treatments—have been developed to combat metastatic progression [3]. However, their clinical efficacy in targeting metastasis remains suboptimal, even when combined with promising immunotherapies. This limitation stems primarily from tumor heterogeneity, the immunosuppressive tumor microenvironment (TME), poor immunogenicity, immune tolerance, and systemic toxicity [4, 5]. Consequently, effective metastasis treatment remains a formidable challenge. Recent advances in deciphering metastatic mechanisms have revealed a paradoxical role of immune cells in interacting with CTCs. Rather than eliminating CTCs, immune cells appear to increase their metastatic potential [6]. Notably, the formation of heterotypic CTC‒leukocyte clusters has emerged as a critical driver of metastatic dissemination. This paradigm-shifting discovery may unlock novel therapeutic strategies to disrupt metastasis-specific vulnerabilities, offering hope for overcoming the current therapeutic plateau.

CTCs are precursors of metastasis in various cancers [7], representing a subgroup of cancer cells that detach from primary or metastatic tumors and enter the bloodstream [8]. During circulation, however, most CTCs succumb to microenvironmental stressors—including immune surveillance, anoikis, oxidative stress, hemodynamic shear forces, and oxygen/nutrient deprivation—resulting in massive apoptotic attrition and a strikingly low probability of successful metastatic colonization [9, 10]. Patients harboring CTCs are more prone to metastasis and have poorer prognoses. CTCs occasionally circulate through the bloodstream alongside nonmalignant cells, including white blood cells (WBCs) [11, 12]. To identify WBCs associated with CTCs, Dr. Nicola Aceto and colleagues at the University of Basel in Switzerland analyzed blood samples from 70 patients with invasive breast cancer and five tumor mouse models. Among these patients, 34 were CTCs positive, with 88% of the CTCs existing as single cells, 8.6% forming tumor cell clusters, and 3.4% forming CTC‒WBC clusters. The proportion of CTC-associated WBCs varied across different mouse models, ranging from 0.05 to 61%. Through single-cell RNA sequencing, researchers reported that the majority of WBCs within clusters were neutrophils, accounting for 85.5–91.7% of WBCs associated with circulating tumor cells in mouse models and up to 75% in patients [13]. When patients with single CTCs or CTC clusters were compared with those with CTC‒neutrophil clusters, researchers reported significantly shorter progression-free survival in patients with at least one CTC‒neutrophil clusters per 7.5 mL of blood. Similar results were noted in experiments where CTC clusters with neutrophils were injected into tumor-free mice: compared with those receiving only CTC clusters or single tumor cells, mice receiving CTC clusters with neutrophils exhibited faster metastatic progression and higher mortality rates [195] .

Neutrophils have been demonstrated to promote tumor growth and metastasis in oncology research [14] (Table 1). Importantly, however, the phenotype and function of neutrophils at tumor sites may vary depending on the tumor type, stage, location, and microenvironment [15]. Furthermore, neutrophils can be classified into distinct subpopulations based on the basis of surface markers, gene expression profiles, and functional characteristics [16]. For example, neutrophils can be categorized into N1 and N2 subsets, where N1 neutrophils exhibit antitumor activity and N2 neutrophils exhibit protumor activity. Consequently, inhibiting tumor metastasis by intervening in the formation of CTC‒neutrophil clusters presents a significant challenge.

**Table 1:** Role of neutrophils in cancer metastasis

Cell	Functional implications in the cancer metastatic cascade	Mechanistic involvement in the cancer metastatic cascade	Mechanism	References
Neutrophil	Promote the formation of metastatic lesions	Angiogenesis	Biosynthesis and secretion of pro-metastatic mediators: BV8, S100A8/9, and MMP-9	[187]
		Metastasis	Secrete TNF-α, TGF-β, and IL-6; interact with CTCs to form CTC-neutrophil clusters; release NETs, NE, and MMP-9	[38, 51, 52, 66, 162]
		Immunosuppressive modulation of T-cell function	Produce or secrete ARG1, ROS, NO, PD-L1, VISTA	[62]
		Exacerbating genomic instability in neoplastic cells	Produce ROS; secrete microRNA miR-23A and miR-155	[188, 189]
		Driving neoplastic cell proliferation	Secrete EGF, HGF, PDGF, HMGB1, IL-1β, NE, MMP-9, PGE2 and IL-1RA	[68, 70, 93, 190–192]
		Immune evasion mechanisms safeguarding CTCs	Express SIRPα	[42]
	Inhibit the formation of metastatic lesions	Direct cytotoxicity	Express or secrete myeloperoxidase (MPO), reactive oxygen species (ROS) such as H_2_O_2_, various proteases, TNF-α, IFN-γ, IL-2, sTRAIL and IL-17	[193, 194]
		Antibody-dependent cell-mediated cytotoxicity	The effectiveness of ADCC in neutrophils is facilitated by the interaction of antibodies with various Fc receptors on their surface, including FcγRI (CD64), FcγRIIa (CD32), FcγRIIIa (CD16a), and FcγRIIIb (CD16b)	[48]
		Recruitment and activation of innate and adaptive immune cells	Neutrophils engage in dynamic interactions with lymphocytes and DCs, either through direct contact or by secreting key cytokines and chemokines such as IL-8 and TNF-alpha and by presenting antigens via MHC class I and II molecules, neutrophils initiate T-cell responses, facilitating a targeted immune attack against tumor cells	[48]

TNF-α, tumor necrosis factor-alpha; TGF-β, transforming growth factor-beta; IL-6, Interleukin-6; CTCs, circulating tumor cells; NETs, neutrophil extracellular traps; EGF, epidermal growth factor; HGF, hepatocyte growth factor; PDGF, platelet-derived growth factor; VISTA, V-domain immunoglobulin suppressor of T-cell activation; ARG1, arginase1; NE, neutrophil elastase; MMP-9, matrix metalloproteinase 9; HMGB1, high mobility group protein B1

In recent years, therapies such as chemotherapy, radiotherapy (RT), gene therapy (GT), photothermal therapy (PTT), photodynamic therapy (PDT), magnetic hyperthermia therapy (MHT), immunotherapy, and other nonmainstream approaches have emerged as primary methods for suppressing tumors and preventing their invasion and metastasis. However, many experimental studies have shown that monotherapies are often ineffective in preventing cancer metastasis and recurrence. For example, when anti-CD47 antibodies are used to inhibit the interaction between CD47 on CTCs and SIRPα on neutrophils, thereby enhancing neutrophil phagocytosis of CTCs, free anti-CD47 antibodies lack specificity and may cause limitations such as red blood cell toxicity and thrombocytopenia [17]. To address these challenges, targeted drug delivery systems (TDDSs), particularly engineered nanoparticles for TDDSs, have been developed and investigated [18]. These systems involve engineering nanoparticles to integrate targeting ligands, such as antibody fragments, aptamers, or peptide molecules, on their surfaces to create active targeting interfaces. Additionally, to overcome the limitations of single-modality cancer therapies, nanoparticles have been designed to function as multifunctional platforms capable of delivering multiple therapeutic agents, enabling integrated treatment regimens [19]. These strategies offer new therapeutic approaches for targeting CTC‒neutrophil clusters during the highly heterogeneous and dynamically spreading process of cancer metastasis, with the aim of inhibiting neutrophil-mediated CTCs metastasis.

While eliminating or suppressing CTCs metastasis may prove unnecessary during early tumorigenesis and exhibit diminished therapeutic returns in advanced stages, targeted CTCs eradication or metastatic suppression retains clinical relevance for specific scenarios—particularly in managing unresectable malignancies and implementing prophylaxis against metastatic recurrence. In this review, we comprehensively summarize nanomaterial-based drugs aimed at modulating the interaction between neutrophils and CTCs. The focus will be on nanomaterial drug delivery systems designed to kill CTCs directly, reprogram neutrophil phenotypes, minimize NETs, reduce neutrophil recruitment to tumor sites, and block the formation of CTC‒neutrophil clusters. Additionally, we discuss future perspectives and challenges regarding nanomaterial-based therapies targeting neutrophil-mediated cancer metastasis.

## General metastasis mechanisms of CTCs

Tumor metastasis relies on premetastatic niche formation, where primary tumors release tumor-derived secretory factors (TDSFs) and extracellular vesicles (EVs) to remodel distant microenvironments via bone marrow-derived dendritic cells (BMDCs) or tissue-resident cells [2, 20, 21]. The premetastatic niche has six critical features—inflammation, immunosuppression, angiogenesis, lymphangiogenesis, organotropism, and reprogramming—facilitating CTCs colonization through purposeful microenvironment priming [22].

EMT is a critical cellular program for malignant tumor progression [23, 24]. When triggered by extracellular molecules (such as TGF-β, hepatocyte growth factor (HGF) and insulin-like growth factor (IGF)) and tumor microenvironment stimuli (such as hypoxia), EMT transforms tumor cells from an epithelial state to a mesenchymal state and endows tumor cells with greater invasion ability and greater metastatic potential, thereby inducing intravasation and shedding of tumor cells. In addition, EMT is directly connected to the gain of mesenchymal and stem cell properties, which enhance the self-renewal and tumor-initiating capabilities of cancer cells [25].

After shedding from the primary tumor, invasive and metastasis-competent tumor cell clones enter the bloodstream as individual CTCs or CTC clusters [26]. Several studies have shown that CTC clusters have greater viability than individual CTCs when facing various death threats [27]. In addition to CTCs themselves, CTC clusters sometimes gather immune cells, platelets and cancer-associated fibroblasts (CAFs) [24, 28, 29]. Notably, neutrophils represent the most abundant leukocyte subset within these clusters. Interactions between CTCs and neutrophils lead to the formation of CTC–neutrophil aggregates, which not only increase CTCs growth and survival but also impair the host's adaptive and innate immune responses [13]. Consequently, targeting or inhibiting the formation of these aggregates has emerged as a promising strategy for the study and treatment of tumor metastasis.

CTCs that metastasize to distant organs may not form metastases immediately. Instead, certain CTCs may enter a state of dormancy before later proliferating under favorable circumstances, ultimately leading to the formation of metastases or tumor recurrence [30, 31].

Recent studies in CTCs have revealed the critical role of the tumor microenvironment in the initiation and progression of metastatic disease [32]. Specifically, tumor-associated cells, such as tumor-associated macrophages (TAMs), CAFs, and BMDCs, help sustain the aggressive tumor microenvironment and metastatic phenotype of CTCs. In return, the direct interaction with or secretion of factors from CTCs accelerate the transformation of tumor-related cells into protumor phenotypes [33, 34].

## CTC–neutrophil crosstalk promotes tumor metastasis through specific interaction mechanisms

**Fig. 1:**
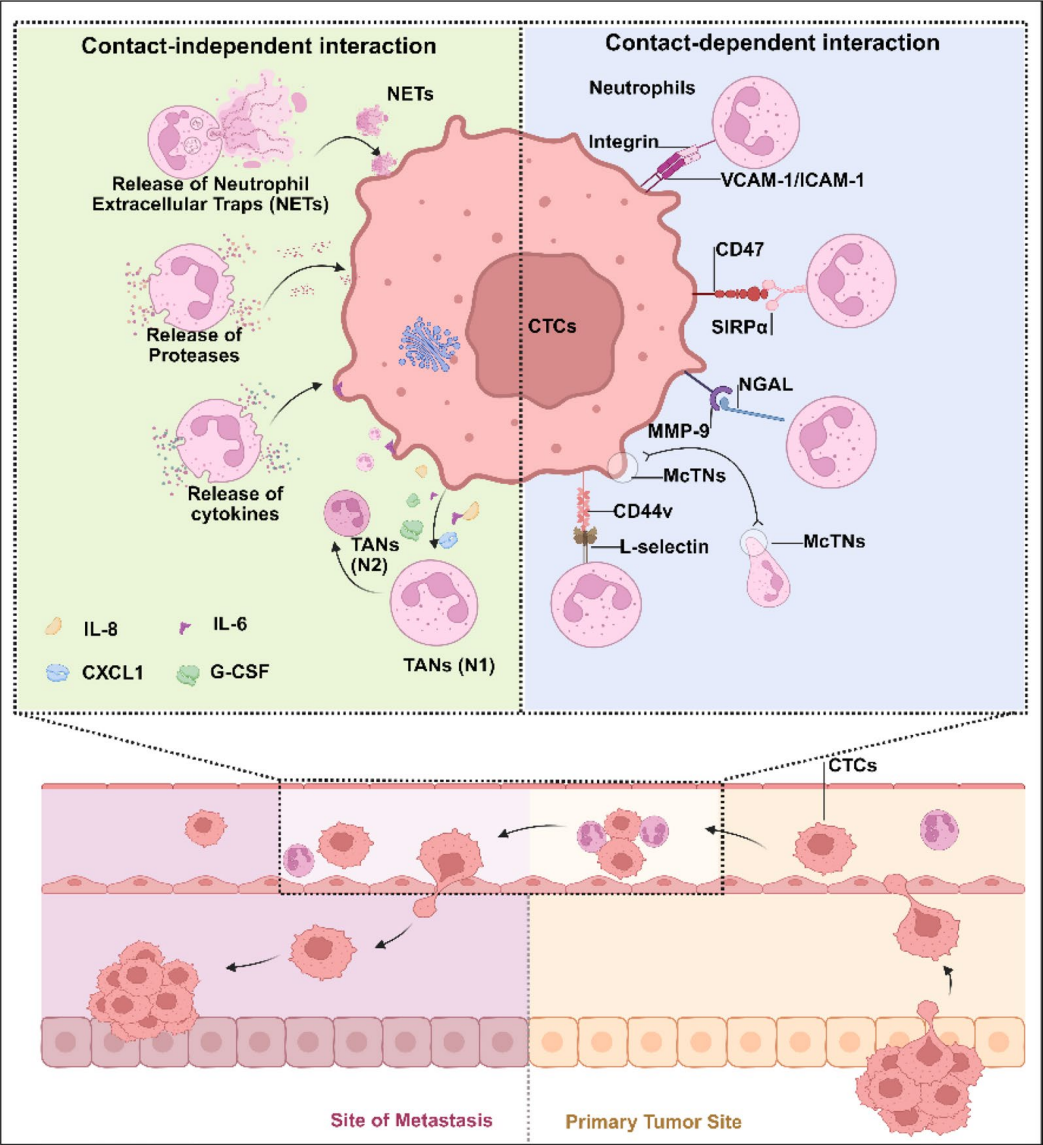
Neutrophils interact with CTCs through both direct and indirect mechanisms. Direct interactions occur when CTCs express ligands such as ICAM-1/VCAM-1, CD47, MMP-9 and CD44v, which bind to neutrophil surface receptors, including integrins, SIRPα, NGAL, and selectins. Additionally, direct interactions can involve the formation of microtentacles (McTNs) between neutrophils and CTCs, leading to the formation of clusters. Indirect interactions are mediated by CTC-secreted cytokines, such as granulocyte colony-stimulating factor (G-CSF), interleukin-6 (IL-6), CXCL1, interleukin-8 (IL-8), tumor necrosis factor-alpha (TNF-α), interleukin-1 beta (IL-1β), and apolipoprotein E (APOE), which regulate the phenotype and activation state of neutrophils to exert protumorigenic effects. Notably, neutrophils themselves secrete various cytokines, including IL-8, G-CSF, TNF-α, TGF-β, IL-6, EGF, HGF, PDGF, IL-1β, VEGF, IL-1RA, and IL-10, which modulate the behavior of CTCs and the tumor microenvironment. Furthermore, neutrophils release diverse proteases, such as neutrophil elastase (NE), cathepsin G (CG), proteinase 3 (PR3), matrix metalloproteinases (MMPs), and arginase 1 (Arg 1), which degrade the extracellular matrix (ECM) and regulate the activity of growth factors, cytokines, and chemokines, facilitating CTCs invasion and extravasation. Additionally, neutrophils can form NETs to indirectly capture CTCs. NETs mask surface antigens on CTCs, protecting them from natural killer (NK) cell-mediated cytotoxicity and macrophage phagocytosis. NETs also activate the TGF-β signaling pathway to induce epithelial‒mesenchymal transition (EMT) in CTCs and release proteases or reactive oxygen species (ROS) to degrade the ECM and increase vascular permeability, thereby promoting CTCs dissemination. Created in BioRender. Su, Y. (2025) https://BioRender.com/h54e244 (accessed on 23 March 2025)

Neutrophils exhibit multifaceted interactions with CTCs, encompassing both direct contact and indirect communication. Direct mechanisms include cell‒cell adhesion and NETs, whereas indirect pathways involve bidirectional paracrine signaling mediated by cytokines, proteases, and chemotactic factors. Notably, CTCs actively secrete chemokines to recruit and activate neutrophils, which reciprocally modulate CTCs behavior through autocrine and paracrine loops. These interactions dynamically regulate critical CTCs functions, such as survival, proliferation, invasion, and extravasation, and profoundly reshape the premetastatic niche and tumor microenvironment. The interplay between neutrophil-derived effector molecules and CTC-driven recruitment signals underscores a complex network of reciprocal regulation, with implications for both metastasis progression and therapeutic targeting. Here, we summarize the current literature on the role of CTC-neutrophil in tumor metastasis, focusing on five aspects: CTC-neutrophil clusters, CTC-neutrophil extracellular traps, CTC-neutrophil cytokines, CTC-neutrophil proteases and CTC cytokines-neutrophils (Fig. 1).

### Contact-dependent interaction between CTCs and neutrophils

#### CTC‒neutrophil clusters

CTCs are commonly found in the bloodstream in aggregated forms, often forming clusters with other CTCs or nontumor cells [35]. Interestingly, CTC clusters present significantly greater metastatic potential than individual CTCs do. This phenomenon is attributed primarily to the enhanced resistance of CTC clusters to apoptosis and immune clearance, as well as their strengthened ability to adhere to endothelial cells and nontumor cells, which promotes their extravasation and colonization at distant sites [36, 37]. Among these nontumor cells, neutrophils are the most frequently observed interacting cell type associated with CTCs [13, 38]. Research has shown that neutrophils play a critical role in the early stages of metastasis by guiding CTCs through the cell cycle. Additionally, neutrophils can protect CTCs from NK cell-mediated cytotoxicity by forming physical barriers and secreting immunosuppressive cytokines [39]. The molecular mechanisms that mediate the formation and function of CTC‒neutrophil clusters are not fully understood, but several possible candidates have been identified. For example, the upregulated intercellular adhesion molecule-1 (ICAM-1) and vascular cell adhesion molecule-1 (VCAM-1) on CTCs can specifically bind to β2 integrin on neutrophils [40, 41]. Additionally, signal regulatory protein α (SIRPα) on neutrophils interacts with CD47 expressed on CTCs [42]. Furthermore, neutrophil-derived neutrophil gelatinase-associated lipocalin (NGAL) can bind to matrix metalloproteinase-9 (MMP-9) on CTCs [43]. Moreover, CTCs expressing ligands such as CD44v can interact with L-selectin on neutrophils, further promoting their mutual association [44]. Recent studies have also identified a novel structure in neutrophils—McTNs, which are composed of vimentin intermediate filaments and posttranslationally modified α-tubulin forms (including acetylated and tyrosine-depleted α-tubulin)—that may play a key role in mediating heterotypic clustering between CTCs and neutrophils [45]. These intermolecular interactions collectively form a complex network governing CTC‒neutrophil interactions, offering critical insights into the dynamic regulation of tumor metastasis.

#### CTC-neutrophil extracellular traps

NETs are extracellular DNA structures released by neutrophils in response to stimuli such as bacteria, fungi, viruses, or inflammatory mediators. These structures are decorated with histones and granular proteins [46]. Recent studies have shown that NETs not only capture pathogens but also interact with CTCs to regulate tumor metastasis [47]. NETosis is categorized into two primary types [48]: suicidal NETosis and vital NETosis. Suicidal NETosis, typically induced by potent microbial infections or robust inflammatory stimuli, including cytokines and chemical agents, involves a coordinated molecular cascade initiated by NADPH oxidase-dependent reactive ROS generation, mitochondrial respiratory burst-derived ROS amplification, protein kinase C (PKC) activation, and peptidyl arginine deiminase 4 (PAD4)-mediated histone citrullination that collectively promotes chromatin decondensation and subsequent extracellular release of NETs concomitant with cellular membrane rupture. In contrast, viable NETosis enables neutrophils to retain membrane integrity through a distinct secretory mechanism, wherein lipopolysaccharide (LPS) engagement with TLR2 on neutrophils and TLR4 on platelets triggers intracellular Ca^2^⁺ signaling that phosphorylates and activates PAD4, leading to NETs packaging within membrane-bound vesicles for extracellular release without cytolysis. Notably, in the tumor microenvironment, tumor-derived stimuli such as granulocyte G-CSF, IL-8, and tumor exosomes trigger ROS-dependent suicidal NETosis via the TLR4 signaling pathway. Concurrently, platelet‒neutrophil interactions drive vital NETosis through TLR2-mediated Ca^2^⁺ signaling [49]. The mechanistic dichotomy of NETosis enables pathway-specific nanomedicine strategies, with ROS-dependent suicidal NETosis inhibited by NADPH oxidase inhibitors (e.g., diphenylene iodonium, DPI) or ROS scavengers targeting NOX2 activity, whereas Ca^2^⁺-dependent vital NETosis is selectively suppressed via TLR2/4 antagonists or PAD4 inhibitors (e.g., GSK484), thereby offering dual therapeutic avenues to modulate pathological NETs formation in inflammatory or tumor microenvironments [50].

However, the impact of NETs on tumor metastasis is dual-sided, with their specific effects depending on the local microenvironment. In some contexts, NETs promote tumor metastasis through multiple mechanisms. For example, amyloid-beta produced by CAFs can induce NETs formation. NETs subsequently degrade the extracellular matrix (ECM) via the release of NE and MMP-9, thereby reactivating dormant cancer cells [51, 52]. Additionally, NETs can form physical barriers and secrete immunosuppressive cytokines, protecting CTCs from NK cell-mediated cytotoxicity [38,53].Furthermore, NETs increase CTCs invasiveness by providing migratory scaffolds and activating MMP-9 [54]. Importantly, NETs facilitate CTCs extravasation by degrading ECM components and increasing vascular permeability. For example, Zhang et al. demonstrated that inhibiting NETs formation via ivermectin (IVM) effectively prevents melanoma metastasis [55], whereas Najmeh et al. reported that DNase 1 reduces CTCs metastatic potential by eliminating cancer cell-NET adhesion [56]. On the other hand, MPO, proteases and histones, among other NETs components, can destroy tumor cells and prevent their development and metastasis. NETs can suppress tumor metastasis by inhibiting CTCs survival and proliferation [57]. According to Shahzad et al*.*, NETs can prevent CTCs from entering the liver and lung blood vessels [58]. In addition, as the tumor environment changes, NETs may promote a Th1-like tumor immune microenvironment by activating peripheral PBMCs, promoting the recruitment of T cells and monocyte-macrophages, preventing tumor growth, and inducing Th1 cell proliferation [59]. By attracting CD8^+^ T cells and preventing regulatory T cells, NETs can also alter the immune response in ovarian cancer, improving overall survival in patients with high-grade ovarian cancer [60]. In summary, the role of NETs in tumor metastasis is highly context dependent, reflecting the complexity of their interactions with tumor cells and the tumor microenvironment. Understanding these mechanisms holds significant promise for the development of innovative therapeutic approaches aimed at controlling tumor progression.

### Contact-independent interaction between CTCs and neutrophils

#### CTC-neutrophil cytokines

Neutrophils are the most abundant type of white blood cell in both blood and tissues. As myeloid-derived cells, they are released from the bone marrow in a terminally differentiated state and serve as first-line responders to inflammation and infection, rapidly reaching sites of inflammation. Notably, peripheral blood neutrophil counts are often abnormally elevated in cancer patients, and the protumor and antitumor effects of neutrophils may stem from their functional diversity under different polarization states [61].

In terms of protumor functions, neutrophils actively participate in tumor progression by secreting various cytokines. For example, IL-8 and G-CSF act as autocrine or paracrine factors, stimulating the proliferation, survival, invasion, and angiogenesis of CTCs [62, 63]; TNF-α induces endomorphosis and stem-like differentiation in CTCs while enhancing their adhesion to endothelial cells [64]; TGF-β promotes endoderm-like transformation and stem-like differentiation in CTCs while suppressing their immunogenicity [65]; IL-6 drives the EMT in CTCs, facilitating tumor cell migration [66]; EGF, HGF, and PDGF activate the MAPK and PI3K/AKT pathways in CTCs, thereby promoting their growth [62, 67]; IL-1β stimulates CTCs proliferation [68]; and VEGF supports tumor metastasis by promoting angiogenesis and vascular permeability in premetastatic niches and tumor microenvironments [69]. Additionally, IL-1RA disrupts the senescence-associated secretory phenotype of tumors, protecting proliferating tumor cells from senescence regulation in a paracrine manner [70]; IL-10 enhances distant metastasis by activating the c-Met/STAT3 signaling pathway [71]. On the antitumor side, neutrophils exhibit multifaceted antitumor functions under tumor microenvironment stimuli, integrating direct cytotoxic mechanisms, antibody-dependent cell-mediated cytotoxicity (ADCC), and immunomodulatory pathways to suppress tumor progression. Direct antitumor effects are primarily mediated by the secretion of cytokines, including IFN-γ, IL-12, TRAIL, and calprotectin, which collectively inhibit tumor growth and metastasis through distinct mechanisms: IFN-γ activates antitumor immune responses to block tumor progression [72]; IL-12 enhances T-cell activation and enhances adaptive immunity against cancer cells [73]; TRAIL induces apoptosis in tumor cells via death receptor signaling [74]; and calprotectin directly restrains tumor cell proliferation and migratory capacity [75]. Simultaneously, neutrophils eliminate tumor cells through the ADCC and orchestrate antitumor immunity by recruiting/activating innate and adaptive immune cells (e.g., NK cells and T cells) [48]. These findings highlight the complex and contradictory roles of neutrophils in the tumor microenvironment, with their functions regulated by multiple factors. Elucidating the mechanisms by which neutrophils influence tumors under different polarization states could pave the way for the development of new therapeutic strategies that target tumor metastasis.

#### CTC-neutrophil proteases

Neutrophils contain four types of granules, within which a series of important proteases are stored, including NE, cathepsin G (CG), proteinase 3 (PR3), matrix metalloproteinases (MMPs), and Arg1, among others [76]. Upon release, these proteases not only remodel the extracellular matrix (ECM) but also play a critical role in the tumor microenvironment by processing and degrading various cytokines, chemokines, and their homologous receptors. Specifically, these proteases exert profound effects on tumor proliferation, vascular density, and metastatic potential. For example, NE modifies lipoproteins, regulates cytokine activity, and modulates MMP activity. Additionally, it degrades elastic proteins, fibrous and nonfibrous collagens, and other ECM components, thereby reshaping the tumor microenvironment [77]. Furthermore, NE enhances the invasiveness of CTCs by cleaving E-cadherin and inducing EMT, significantly increasing CTCs invasiveness [78, 79]. NE also disrupts endothelial cell junctions, increasing vascular permeability and promoting CTCs extravasation [80]. MMP-9 contributes to CTCs invasion by degrading the basement membrane and activating pro-MMP-2 [81]. Additionally, MMP-9 induces the expression of vascular endothelial growth factor receptor 1 (VEGFR1) in endothelial cells, further facilitating CTCs extravasation [82]. Notably, Arg 1 indirectly promotes tumor progression by depleting extracellular arginine and inhibiting T-cell function [83]. These mechanisms highlight the complex and pivotal role of neutrophil-derived proteases in tumor metastasis.

#### CTC cytokines-neutrophils

CTCs are capable of secreting multiple cytokines, some of which may exert protumorigenic effects by regulating the phenotype and behavior of neutrophils. The key cytokines involved in this process include G-CSF, IL-6, CXCL1, IL-8, TNF-α, IL-1β, and APOE. G-CSF plays a significant role in reprogramming neutrophils into a tumor-promoting phenotype [84]. IL-6 activates the STAT3 signaling pathway in neutrophils [85], driving their polarization from the N1 phenotype to the N2 phenotype and thereby enhancing their tumor-promoting effects [86]. CXCL1 interacts with its receptors CXCR1 or CXCR2 to recruit neutrophils [87, 88], a process that facilitates their recruitment and activation within the tumor microenvironment and circulation [89]. Similarly, IL-8 attracts neutrophils and upregulates the expression of Mac-1 on their surface, enhancing their anti-shear adhesion ability with tumor cells expressing ICAM-1 [40]. Additionally, TNF-α and IL-1β stimulate the NF-κB signaling pathway in neutrophils, leading to the expression of proinflammatory genes and the production of reactive ROS [90]. Notably, APOE on the surface of CTCs interacts with TREM2 on neutrophils, triggering an aging process in these cells. These senescence-like neutrophils secrete a range of bioactive molecules collectively referred to as the senescence-associated secretory phenotype (SASP). These molecules create an immunosuppressive microenvironment, inhibit the activation of NK cells and cytotoxic T lymphocytes, and promote chronic inflammation and tumor progression [91]. Furthermore, CTCs induce the production of endogenous Toll-like receptor (TLR) 2/4 ligands, such as S100A8, S100A9, and SAA3. These ligands may amplify the expression of proinflammatory cytokines, further influencing neutrophil function [92]. In conclusion, CTCs exert multifaceted effects on neutrophils through the secretion of various cytokines, shaping their phenotype and behavior in ways that promote tumor progression. These findings provide valuable insights into the complex interplay between CTCs and neutrophils, offering potential therapeutic targets for combating cancer.

#### Others

Neutrophils secrete prostaglandin E2 (PGE2), a nutritional signaling molecule, which significantly increases the number of precancerous lesion cells in injured caudal fin tissue [93]. Additionally, pulmonary mesenchymal cells (MCs) induce lipid storage within neutrophils and provide essential nutritional support to disseminated tumor cells [94]. These findings are further supported by research demonstrating that neutrophils express transferrin, an iron-transporting protein, at both the mRNA and protein levels in both murine and human neutrophils. Transferrin primarily mediates the mitogenic activity of neutrophils on tumor cells. Furthermore, in the metastatic microenvironment, tumor cells produce large amounts of granulocyte‒macrophage colony‒stimulating factor (GM-CSF), which specifically targets neutrophils via activation of the Jak/Stat5β pathway, thereby upregulating transferrin gene expression [95].

In summary, the interaction network between CTCs and neutrophils significantly enhances the survival, proliferation, and metastatic potential of CTCs. Targeting the formation of CTC‒neutrophil complexes is considered a highly promising therapeutic strategy to suppress tumor metastasis. Research by Liu et al. has demonstrated that anti-ICAM1 neutralizing antibodies, by blocking ICAM1 expression, significantly inhibit the aggregation of CTCs with neutrophils, effectively curbing neutrophil-mediated metastasis of triple-negative breast cancer (TNBC) [96]. However, from a clinical perspective, free-form anti-ICAM1 antibodies may interfere with multiple intercellular interactions during tumor development and metastasis, potentially impairing normal immune functions that depend on the interaction between LFA-1/Mac-1 and ICAM-1. Therefore, developing methods to precisely target ICAM-1 on CTCs, thereby minimizing adverse effects caused by off-target effects, represents significant research value. This approach not only holds promise for improving therapeutic efficacy but also reduces interference with normal physiological functions, offering a novel avenue for controlling tumor metastasis.

## Nanotherapeutics that suppress CTC‒neutrophil cluster formation

Although targeting CTC‒neutrophil complexes and their associated NETs may represent a promising strategy to disrupt tumor dissemination and progression, their practical application remains fraught with challenges. Owing to the high heterogeneity and rarity of CTCs, as well as the complexity of NETs, conventional therapeutic approaches may be limited in terms of efficacy and specificity. In this context, nanomedicine, as a significant application of nanotechnology in the medical field, offers a novel solution to overcoming these challenges by delivering therapeutic agents with high selectivity to target cells and tissues [97, 98]. Therefore, this study focuses on reviewing recent advancements in various nanomedicine platforms that target CTCs elimination, reduce NETs formation, reprogram neutrophil phenotypes, inhibit neutrophil recruitment to tumor sites, and directly block neutrophil‒CTC clusters formation.

### Direct therapeutic targeting of CTCs

CTCs secrete various cytokines, some of which may exert tumor-promoting effects by modulating the phenotypic and behavioral profiles of neutrophils. These include G-CSF [84], IL-6 [85], CXCL1 [87, 88], IL-8 [40], APOE [91], TNF-α and IL-1β [90]. Furthermore, CTCs can form clusters with neutrophils via intercellular adhesion molecules, thereby enhancing CTCs metastasis [40, 41]. This therapeutic strategy focuses on exploiting specific surface markers or metabolic signatures of CTCs to achieve targeted delivery of cytotoxic agents, thereby reducing CTC‒neutrophil interactions. Given that approximately 90% of human cancers originate from epithelial cells [99], EpCAM has emerged as the most commonly utilized marker for CTCs detection and targeting [100]. Furthermore, numerous studies have identified additional biomarkers, such as human epidermal growth factor receptor 2 (HER2) [101], estrogen receptors [102], and folate receptors [103], which serve as characteristic markers for CTCs across various types of cancers. Notably, tumor cell membranes also highly express CD44 receptors [196], integrin receptors [104], ICAM-1 [96], and VCAM-1 [105]. These findings provide a rich array of targetable sites for nanotherapeutic agents aimed at eliminating CTCs. Building on these targets, this review will detail strategies to disrupt CTC‒neutrophil clusters formation by focusing on two distinct nanotherapeutic approaches: biological membrane-modified nanosystems that utilize cell membrane coatings for targeted CTCs recognition and killing and nonmembrane-based nanoplatforms that exploit molecular ligands or metabolic vulnerabilities specific to CTCs. These classifications highlight how nanotechnology can leverage CTCs surface features to eliminate CTCs while impeding their prometastatic interactions with neutrophils.

#### Biological membrane-modified nanosystems for targeted CTC recognition and killing

##### 1) Neutrophil membrane-camouflaged nanoplatforms for CTC-targeted eradication

**Fig. 2:**
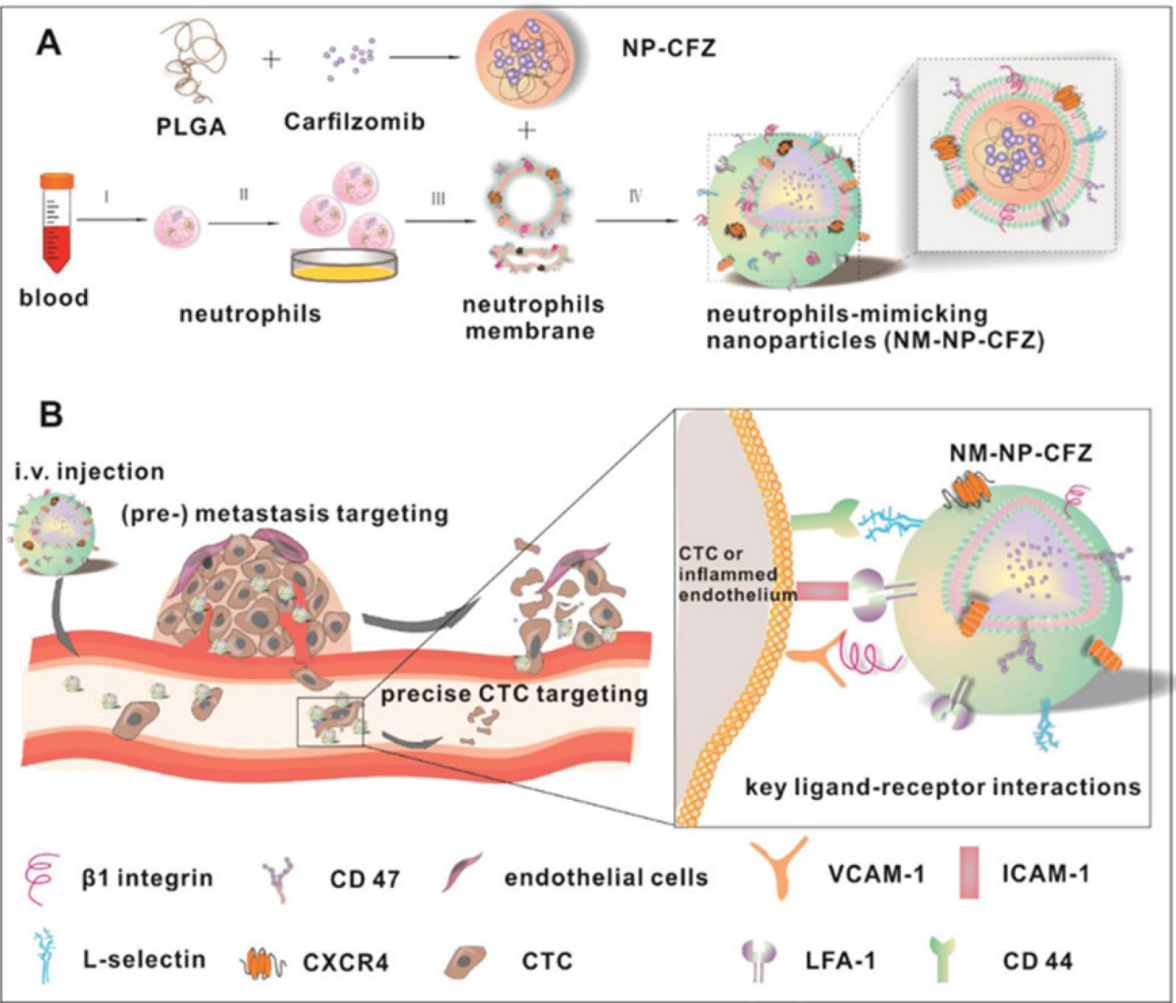
Schematic of NM-NP-CFZ depleting CTCs and colonization sites. **A** Scheme for the preparation of NM-NP-CFZ. I. Neutrophils were extracted from whole blood via the Percoll® gradient separation method. II. Isolated neutrophils were stimulated with LPS. III. Centrifugation separated the plasma membrane of the LPS-stimulated neutrophils. IV. NM-NP-CFZ was prepared by coating the plasma membrane of LPS-stimulated neutrophils with PLGA nanoparticles. The mixture of neutrophil membrane-associated proteins enables the resulting NM-NP CFZ to target CTCs in circulation and inflamed endothelial cells in premetastatic lesions. Three pairs of key interactions, including the binding of LFA-1 with ICAM-1, CD44 with L-selectin, and β1 integrin with VCAM-1, are involved in the CTCs and inflamed endothelium-targeting of NM-NPs. Reproduced with permission from Kang et al. (2017). Copyright^©^ 2017 American Chemical Society

Given the abundance of neutrophils in the peripheral blood and their critical role in cancer metastasis and recurrence, they have been extensively studied and proposed as natural drug carriers for cancer therapy [106, 107]. As one of the key events in the early stages of tumor metastasis, the colonization of CTCs in premetastatic niches is considered a highly promising therapeutic target. Research has shown that inflammatory neutrophils can recognize and bind CTCs expressing specific adhesion molecules while also targeting premetastatic niches [108]. Kang et al. developed an innovative nanodrug delivery system by coating poly (lactic-co-glycolic acid) polymer nanoparticles (NPs) with inflammatory neutrophil membranes (NM) and loading the proteasome inhibitor carfilzomib (CFZ) into the nanoparticles, forming NM-NP-CFZ nanodrugs. This nanodrug retains key adhesion proteins on the neutrophil surface, such as integrin family members, while also inheriting various proteins capable of specifically recognizing CTCs and targeting premetastatic niches. Through the loading of CFZ, the nanodrug can precisely target CTCs: on the one hand, CFZ induces apoptosis in CTCs by inhibiting proteasome function; on the other hand, it significantly suppresses the proliferative capacity of CTCs. This dual mechanism enables NM-NP-CFZ to effectively eliminate circulating CTCs, thereby preventing early tumor cell dissemination to distant organs such as the lungs and potentially inhibiting established metastases [109] (Fig. 2).

##### 2) Exosome membrane-camouflaged nanotherapeutics for CTC-specific ablation

**Fig. 3:**
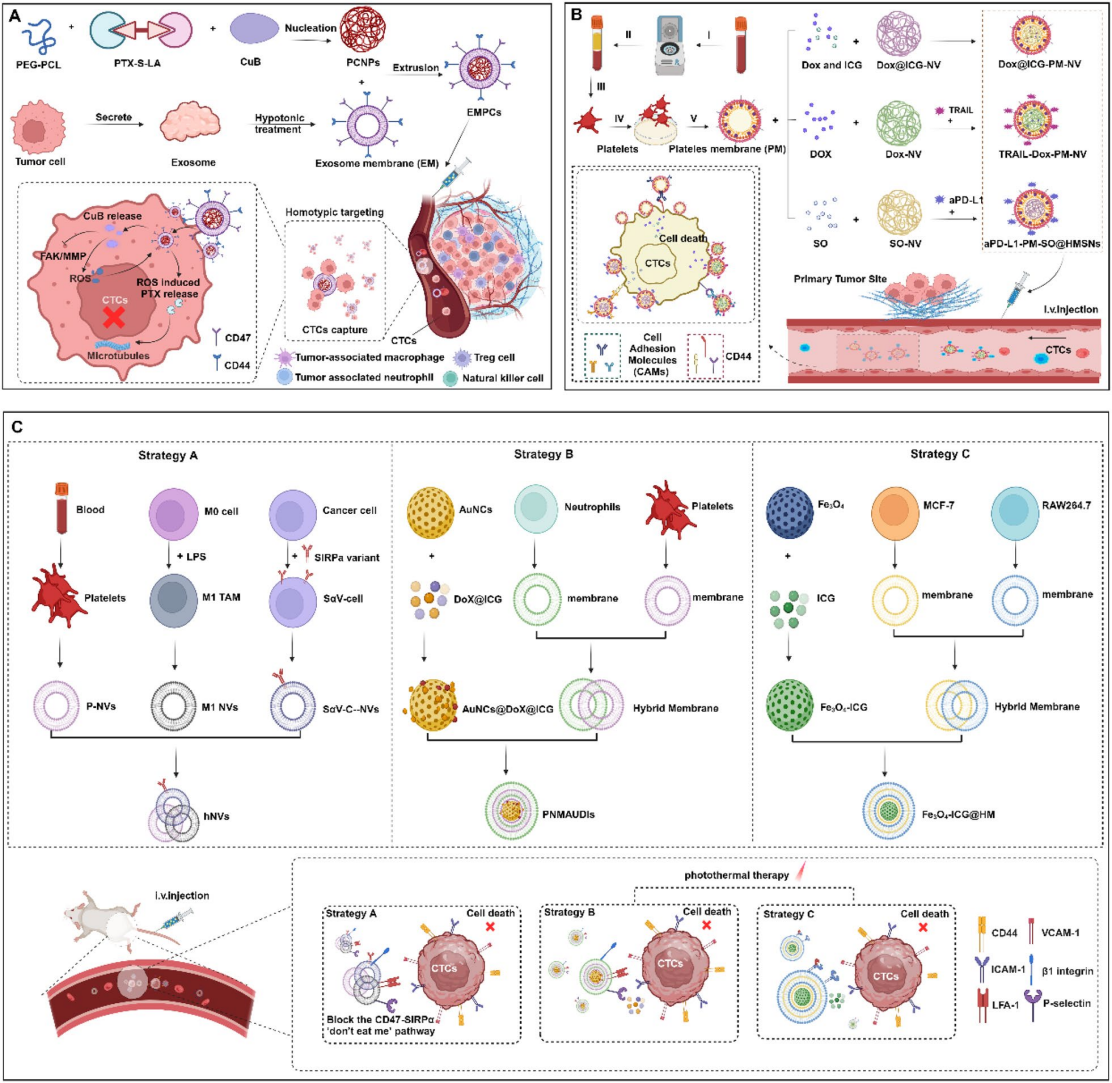
Nanomaterials for targeted killing of CTCs. **A** Schematic of EMPCs for eliminating CTCs. EMPCs, which are made by coating nanoparticles with exosome membranes from tumor cells, bind CTCs via homotypic recognition. In the tumor microenvironment, elevated ROS levels trigger the release of cucurbitacin B (CuB), which inhibits metastatic signaling and induces CTCs apoptosis. **B** Schematic of the use of PM-NPs for CTCs elimination. PM-NPs use platelet membrane CAMs (e.g., P-selectin) to target CTCs. They are loaded with chemotherapeutic agents (e.g., DOX, sotorasib, TRAIL) or immune checkpoint inhibitors (aPD-L1) to induce CTCs apoptosis or enhance antitumor immunity. PM-NPs also encapsulate ICG for NIR light-triggered hyperthermia and immunogenic cell death. **C** Schematic of fusion membrane nanodrugs/vesicles that target CTCs. These constructs use membranes from platelets, M1 macrophages, tumor cells, and leukocytes to evade immune clearance and target CTCs. For example, hNVs fuse P-NVs, M1-NVs, and SαV-C-NVs to interact with CTCs, block the CD47-SIRPα pathway, and stimulate macrophage phagocytosis. PNMAuDIs use platelet and neutrophil membranes to target CTCs and release DOX and ICG locally. Fe3O4-ICG@HM uses hybrid membranes to interact with tumor cells and provides magnetic and photothermal therapy to eliminate CTCs. Created in BioRender. Su, Y. (2025) https://BioRender.com/h54e244 (accessed on 23 March 2025)

Exosomes are a class of EVs with diameters ranging from 50 to 150 nm that are secreted by various cell types, including cancer cells, immune cells, stem cells, and endothelial cells [110]. Cancer-derived EVs show natural tropism for both primary tumors and metastatic sites and can also target tumor-associated immune cells [111]. In recent research on engineering tumor-derived EVs to interfere with CTC-neutrophil interactions, significant progress has been made [111]. Researchers have identified multiple specific surface adhesion molecules, such as CD44 and CD47, in exosomal membranes. CD44 mediates homotypic targeting, whereas CD47 interacts with integrin-associated proteins to assist cancer cells in evading macrophage-mediated phagocytosis within the immune system [112, 113]. Leveraging these properties, Wang et al. developed a nanodrug modified with exosomal membranes (EMPCs), which specifically captures and neutralizes CTCs in circulation through high-affinity interactions. The design of EMPCs integrates two key components: a reactive ROS-sensitive thioether-linked paclitaxel-oleate prodrug (PTX-S-LA) and the tetracyclic triterpenoid molecule cucurmin B (CuB). Upon entering tumor cells, CuB is released and significantly inhibits tumor metastasis by downregulating the FAK/MMP signaling pathway. Additionally, CuB elevates intracellular oxidative levels, triggering the sequential bioactivation of ROS-sensitive PTX-S-LA. The experimental results demonstrated that EMPCs exhibit enhanced circulation time, selective targeting of homologous tumor cells, and superior tumor penetration both in vitro and in vivo. Furthermore, EMPCs effectively suppress tumor metastasis by eliminating CTCs and regulating the FAK/MMP signaling pathway [114] (Fig. 3A). This work represents a significant step forward in understanding how to utilize tumor-derived EVs to disrupt the interaction between CTCs and neutrophils, potentially providing new strategies for preventing and treating tumor metastasis.

##### 3) Platelet membrane-engineered nanovehicles for CTC-specific elimination

Platelet play pivotal roles in tumor–tumor cell adhesion and communication due to the presence of cell adhesion molecules (CAMs) on their membrane surfaces [115]. Platelet membrane-wrapped nanoparticles (PNPs) hold significant potential in cancer therapy, as the platelet membrane serves as an effective drug delivery carrier while maintaining fundamental platelet characteristics, such as surface molecules and proteins. In the context of cancer, the overexpression of P-selectin receptors on the platelet membrane aligns with the upregulation of CD44 receptors on cancer cells, enabling these nanoparticles to more precisely target cancer cells and enhance drug delivery efficiency [116, 117]. Ye et al. decorated NP surfaces with platelet membranes (PMs) and encapsulated the chemotherapeutic agent doxorubicin (DOX) and the photothermal agent indocyanine green (ICG) within bioengineered nanoplatelets. This system efficiently inhibited breast cancer metastasis in xenograft or orthotopic breast tumor-bearing mouse models. The bioengineered nanoparticles leverage the high-affinity interaction between PM-bound P-selectin and tumor cell CD44 receptors to specifically capture and clear CTCs in the blood and lymphatic circulation, significantly suppressing tumor metastasis [118]. Hu et al. loaded TRAIL and DOX into PM-modified core‒shell nanocarriers (PM‒NVs), creating TRAIL‒Dox‒PM‒NV nanodrugs for targeted tumor therapy. P-selectin, which is overexpressed on the PM, specifically interacts with upregulated CD44 receptors on CTCs, facilitating targeted drug delivery. TRAIL induces tumor cell apoptosis by binding to death receptors (DR4 and DR5) on tumor cell surfaces [119, 120], whereas DOX embeds itself into cancer cell nuclear DNA to trigger intrinsic apoptotic signaling pathways [121]. Li et al. inspired this process through the adhesion of activated platelets to CTC-associated microthrombi and the conjugation of tumor necrosis factor-related apoptosis-inducing ligand (TRAIL) with activated platelet membrane-coated silica (SiO_2_) particles. This nanodelivery system exploits the affinity of CTCs for activated platelets and immune cell cytotoxicity to achieve targeted therapeutic effects. Furthermore, synthetic particles disguised with platelet membranes were incorporated into thrombi induced by CTCs, enabling localized delivery of anticancer drugs within CTC-containing thrombi and enhancing therapeutic efficacy [122]. Additionally, Da et al. loaded sorafenib (SO), a next-generation multitargeted antitumor drug [123], and aPD-L1 onto hollow mesoporous silica nanoparticles (HMSNs) and coated them with PMs, creating a PD-L1 monoclonal antibody-loaded platelet membrane-coated nanodrug (aPD-L1-PM-SO@HMSNs). This bioengineered nanodelivery system harnesses the interaction between CTCs and platelets to accurately deliver loaded targeted drugs and immune checkpoint inhibitors to cancer cell thrombi (CTC‒platelet clusters) in the circulatory system, effectively killing CTCs [124] (Fig. 3B).

##### 4) Macrophage membrane-engineered nanotherapeutics for CTC-targeted elimination

Macrophages play a significant role in tumor progression and metastasis. The unique α4 integrin property of VCAM-1 enables its interaction with VCAM-1 on cancer cell surfaces, making it an attractive carrier for treating lung metastasis [125]. In a breast cancer lung metastasis model, Li et al. developed an innovative nanodelivery system by embedding pH-sensitive Dox-MPK precursor drugs into a DNA-based cellular scaffold, followed by sequential coating with liposomes and macrophage membranes, forming a cell-like Dox-MPK@MDL nanoparticle. This system inherits macrophage-derived metastasis-homing effect-related proteins, enabling specific targeting of breast cancer lung metastatic sites. Additionally, Dox-MPK@MDL nanoparticles can selectively disintegrate at pathological sites, triggering Dox release to directly eliminate CTCs and significantly inhibit breast cancer lung metastasis [126].

##### 5) Hybrid membrane-engineered nanoplatforms for CTC-targeted eradication

Cancer cells express CD47, a "do not eat me" signal, which interacts with signal regulatory protein alpha (SIRPα) on macrophages to prevent phagocytosis [127]. Additionally, platelet-derived vesicles (P-NVs) can interact with CTCs in the bloodstream and bind to damaged vasculature and tissues [128]. Furthermore, vesicles derived from M1 macrophages (M1-NVs) have the potential to reprogram TAMs toward the M1 antitumor phenotype [129]. Inspired by these biological principles, Rao et al. constructed hybrid nanovesicles (hNVs) composed of three fused membranes: P-NVs, M1-NVs, and neoplastic cell-derived vesicles overexpressing a high-affinity SIRPα variant (SαV-C-NVs). Research has demonstrated that hNVs, which inherit the functional capabilities of their parent cells, effectively accumulate at surgical wound sites, interact with CTCs in the bloodstream, and promote TAMs polarization toward the M1 antitumor phenotype. Moreover, hNVs block the CD47-SIRPα interaction, enhancing macrophage-mediated cancer cell phagocytosis and boosting antitumor T-cell immunity. In a melanoma model, intravenous administration of hNVs was shown to control local recurrence and distant metastasis postsurgery, significantly extending the overall survival time of mice [127] (Fig. 3C).

Platelets and neutrophils are recruited to tumor cell surroundings, where they form "early metastatic niches" that promote tumor metastasis. They facilitate the dissemination of CTCs in premetastatic niches and contribute to inflammation-associated metastatic processes [130, 131]. Additionally, these cells form neutrophil/platelet-encapsulated CTCs aggregates, providing protection for circulating CTCs against host immune attacks and physical stresses [132]. Inspired by the natural ability of platelets and neutrophils to target CTCs, as well as the similarities in surface receptors between tumor cells and tumor-derived exosomes, Ye et al. successfully engineered a hybrid cell membrane (PNM) derived from platelets and neutrophils to camouflage gold nanocages (AuNCs). This system was integrated with a photothermal/chemotherapy combination strategy, resulting in the PNM-AuDIs nanodelivery system. The PNM surface is densely populated with adhesive molecules, such as P-selectin and L-selectin, which enable high-affinity interactions with glycoproteins and glycolipids on the surfaces of CTCs and exosomes. Moreover, AuNCs loaded with DOX and ICG effectively eliminate CTCs and tumor-derived exosomes, disrupting the communication between exosomes and immune cells and thereby inhibiting tumor metastasis [133]. (Fig. 3C).

Camouflaging nanoparticles with cancer cell membranes enables them to bind to homologous cancer cells [134]. Coating nanoparticles with WBC membranes imparts surface features that are "homologous" to those of the source WBCs. These homologous features reduce binding between WBCs and nanoparticles [135]. Sun et al. developed an innovative approach integrating bioinspired cell membrane-modified magnetic nanoparticles with an inverted microfluidic chip, achieving high-efficiency and high-purity capture of CTCs while enabling in situ inactivation of captured CTCs. The magnetic nanoparticles were loaded with ICG and further modified with a hybrid membrane composed of human breast cancer cell membranes and WBC membranes. The tumor cell membrane enhanced the material's target specificity, improving capture efficiency, whereas the WBC membranes coating reduced interference from homologous white blood cells, further increasing capture purity. Additionally, polymer photonic crystals were introduced as the CTCs capture interface, providing favorable surface structures to promote CTCs adhesion. After capturing CTCs, the ICG molecules facilitated enhanced photothermal and photodynamic synergetic effects, directly inactivating the captured CTCs [136] (Fig. 3C).

*Rational engineering of biomimetic nanoparticles for CTCs targeting*

To optimize biomimetic nanoparticles for CTCs targeting, critical design parameters must be carefully considered across membrane selection, size engineering, core material design, and surface charge modulation.

Cell membrane sources should prioritize surface protein profiles that mimic CTCs adhesion mechanisms. Neutrophil membranes, for example, inherit integrins (LFA-1, β1) and CD44 to bind CTC-expressed ICAM-1, VCAM-1, and L-selectin, enabling homologous targeting [137]. Hybrid membranes combining CTCs and leukocyte membranes further increase specificity while reducing off-target interactions with blood cells [136]. Red blood cell (RBC) membranes, although lacking intrinsic CTCs affinity, can be functionalized with ligands such as folate or RGD peptides via lipid insertion for active targeting, while their CD47 "do not eat me" markers prolong circulation.

Size optimization is crucial for ensuring the integrity and cellular internalization of the membrane coating. NPs with diameters of 50–100 nm exhibit optimal membrane wrapping efficiency due to their balanced bending energy and ligand‒receptor interactions [138, 139]. Particles < 50 nm face challenges in achieving full membrane coverage, whereas those > 100 nm may induce excessive membrane strain, leading to partial coating [139, 140]. For CTCs targeting, a size range of 50–80 nm balances prolonged circulation (via reduced macrophage uptake) and efficient endothelial extravasation [141].

Core material selection must align with drug-loading requirements and biocompatibility. PLGA remains the gold standard owing to its biodegradability and controlled release kinetics, as demonstrated in neutrophil membrane-coated NPs loaded with carfilzomib [109, 142]. Mesoporous silica or magnetic Fe₃O₄ cores enable multimodal functionalities (e.g., imaging, hyperthermia) while maintaining membrane adhesion [143, 144]. Surface modifications, such as PEGylation, enhance stealth properties but require charge balancing; excessively negative zeta potentials (− 30 mV) improve stability but may hinder CTCs membrane interactions, whereas slightly negative charges (− 10 to − 20 mV) optimize both circulation and tumor accumulation [145].

In summary, successful CTC-targeting biomimetic NPs require the following: (1) membrane customization based on adhesion protein profiles (e.g., neutrophil integrins, hybrid CTC-leukocyte membranes); (2) size control within 50–100 nm to ensure coating integrity and efficient internalization; (3) core material engineering for drug loading and stimuli-responsive release (e.g., PLGA, mesoporous silica); and (4) charge modulation to balance circulation stability (− 10 to − 20 mV) and microenvironment-triggered targeting. These principles, supported by quantitative membrane integrity assessments (> 50% coating for individual uptake; < 50% requiring aggregation), provide a roadmap for advancing CTC-specific nanotherapeutics.

#### Targeted elimination of CTCs using non-biomembrane-coated nanotherapeutics

This strategy primarily employs characteristic surface proteins of CTCs as targeting receptors. The methodology involves either loading corresponding ligands onto nanomaterial surfaces or engineering magnetic nanoparticles to enhance the capture and elimination of CTCs.

##### 1) Targeting E-selectin (Es) on the surface of CTCs

CTCs originating from colorectal, breast, prostate, and pancreatic cancers, among other types, exhibit glycosylated ligands on their surfaces, enabling them to adhere to Es under physiological shear flow conditions [146]. Leveraging this biological characteristic, Ortiz-Otero et al. developed a liposome therapeutic agent modified with E-selectin and TRAIL that achieved significant therapeutic effects in prostate cancer (PC) models. The experimental results demonstrated that this therapeutic agent could kill more than 75% of CTCs in blood samples from prostate cancer patients. However, under identical experimental conditions, the use of soluble TRAIL alone (at a concentration of 290 ng/mL) failed to achieve the expected cytotoxic effect on CTCs. This discrepancy was attributed to the absence of Es-mediated delivery, as E-selectin facilitates the tethering of TRAIL to CTCs surfaces via a membrane-tethering mechanism, thereby increasing its therapeutic efficacy [147]. In subsequent studies, Wayne et al. fabricated liposomes modified with both Es and TRAIL to prevent hematogenous metastasis in prostate cancer. Upon injection into the bloodstream, these nanoparticles selectively adhere to peripheral blood leukocytes, exerting potent cytotoxic effects on circulating cancer cells. Both in vitro and in vivo experiments confirmed the effectiveness of Es/TRAIL liposomes in eliminating CTCs. Specifically, in DU145 cancer cell lines, the presence of Es/TRAIL liposomes in blood samples led to significant apoptosis of cancer cells. In a mouse model of prostate cancer, mice treated with Es/TRAIL liposomes presented a 94% reduction in CTC counts compared with those of buffer-treated controls, further corroborating the remarkable ability of this nanotherapeutic to target and eliminate CTCs [148]. Mitchell et al*.* further optimized this approach by developing a liposomal nanotherapeutic that targets CTCs in colorectal and prostate cancers. This nanotherapeutic was surface-modified with both Es and TRAIL and could bind to circulating leukocytes, forming "functionalized leukocytes," while remaining nontoxic to the leukocytes themselves. Experimental data demonstrated that this nanotherapeutic exhibited exceptional efficacy in treating CTCs in flowing human blood samples and in the peripheral circulation of mice [149]. Moreover, Mitchell et al. engineered a novel nanotherapeutic comprising halloysite nanotubes (HNTs) and Es-functionalized liposomal DOX, designated Es-PEG L-DXR. This nanostructure employs surface modification strategies to enhance the recruitment of chemotherapy to CTCs while simultaneously preventing nonspecific adhesion to healthy cells and off-target uptake of therapeutic agents [150]. Collectively, these studies provide critical theoretical and practical insights into Es-based targeted therapeutic strategies for combating CTCs and metastatic cancer.

##### 2) Targeting epithelial cell adhesion molecule (EpCAM) on the surface of CTCs

Recent mechanistic studies have revealed that the RNA aptamer Ep23 is a high-affinity molecular probe that targets EpCAM, demonstrating dual functionality in both selectively binding to EpCAM-expressing human carcinoma cells and facilitating efficient receptor-mediated endocytosis [151]. By building upon this molecular recognition system, Yao et al. engineered a dual-targeting nanoplatform (dTNP) through covalent conjugation of the K237 peptide (targeting tumor neovasculature) and Ep23 aptamer (targeting CTCs) onto paclitaxel (PTX)-loaded nanoparticles via EDC/NHS chemistry. The K237 peptide mediates high-specificity binding to KDR/Flk-1 tyrosine kinase—a VEGF receptor overexpressed in the tumor vasculature—while the Ep23 aptamer enables CTCs recognition through EpCAM interactions. Functional validation in 4T1-GFP-induced pulmonary metastasis models demonstrated the capacity of dTNPs to intercept CTCs in circulation and neutralize metastatic seeding, as evidenced by intravital flow cytometry, real-time imaging, and confocal microscopy analyses. Notably, this bifunctional targeting strategy achieves synergistic suppression of vascularized tumors and circulating metastatic cells through spatiotemporal drug release, highlighting its potential as a precision therapeutic against hematogenous dissemination [152]. This study exemplifies the rational integration of vascular and cellular targeting moieties to combat metastatic progression at multiple pathological checkpoints.

##### 3) Targeting glucose transporter 1 (GLUT1) in CTCs: disrupting metabolic adaptations for metastatic suppression

Substantial evidence has demonstrated that ginsenoside Rg3, an amphiphilic compound characterized by a hydrophilic glycosyl group and a cholesterol-like lipophilic steroidal structure, has a specific binding affinity for GLUT1, which is overexpressed on tumor cell surfaces [197]. This molecular interaction holds particular therapeutic importance given the crucial role of GLUT1 in basal glucose uptake and its established association with poor clinical prognosis across various solid malignancies, including TNBC, hepatocellular carcinoma, pancreatic ductal adenocarcinoma, esophageal carcinoma, glioblastoma, renal cell carcinoma, non-small cell lung cancer, cutaneous melanoma, colorectal adenocarcinoma, endometrial carcinoma, ovarian cancer, and cervical neoplasms [153]. Building upon this mechanistic foundation, Xia et al. engineered an innovative Rg3-functionalized liposomal system (Rg3-Lp/DTX) for the codelivery of docetaxel (DTX) in TNBC metastasis management. The surface-exposed Rg3 moiety enables active targeting of CTCs through GLUT1-mediated recognition, while the synchronized delivery of Rg3 and DTX achieves dual therapeutic effects: (1) maximal cytotoxic impact of DTX on CTCs populations and (2) preservation of the regulatory capacity of Rg3 over metastatic niche (MN) microenvironment modulation [197].

##### 4) Targeted therapy against CTCs via CD44v6-specific delivery

Notably, among various CD44 isoforms, CD44v6 is expressed specifically in malignant cells, and its presence in patient tumor samples strongly correlates with metastatic progression and poor prognosis [154]. Capitalizing on this molecular signature, Andrade et al. developed CD44v6-targeted polymeric micelles loaded with niclosamide (NCS) for colorectal cancer (CRC) treatment. NCS, an anthelmintic drug repurposed for its anticancer properties, exhibits particular efficacy against cancer stem cells (CSCs) and metastatic malignancies. Experimental evidence confirms that these functionalized polymeric micelles effectively accumulate at CRC-CSC sites and demonstrate potent CTCs eradication capabilities in vivo [155]. In parallel, Gener et al. engineered zileuton-encapsulated polymeric nanoparticles, which utilize this 5-lipoxygenase inhibitor to block leukotriene synthesis, thereby attenuating inflammatory responses and tumor metastasis [156]. In orthotopic tdTomato + MDA-MB-231 models—which represent a highly aggressive, chemotherapy-resistant basal-like breast cancer subtype—PM-Zileuton treatment achieved complete CTCs elimination alongside a reduction in the CSC population. Importantly, no CTCs were detected in the tdTomato + MCF7-ALDH1A1 model (a luminal breast cancer lineage) under identical therapeutic conditions [157].

##### 5) Targeting integrin α5 (ITGA5) on CTCs

ITGA5 is highly expressed in TNBC cells with strong migratory and invasive properties, as well as in their lung metastases [158]. Yang et al. created a nanoparticle-delivered drug named uPtD NPs, which consists of a novel ultrasmall Pt (II) dot separated from miriplatin and loaded in nanoparticles modified with an active ITGA5 antibody. The cell cycle arrest induced by uPtD NPs leading to DNA damage is unique to CSC-like cells in CTC clusters and ultimately suppresses lung metastasis in TNBC [159].

##### 6) Magnetic nano-enhanced enrichment and targeting of CTCs

While PDT-based targeting of CTCs in vitro has demonstrated potential, its clinical translation remains limited by a narrow therapeutic window [160]. To address this critical limitation, Chiang et al. engineered a targetable photosensitive-magnetic core-satellite nanodrug (TCSN) to amplify photoinduced cytotoxicity through synergistic EpCAM-targeted enhancement. The TCSN architecture integrates (i) a magnetic nanocore (MNC) comprising multiple iron oxide nanoparticles (IONs) that enable magnetic enrichment of CTCs under external fields and (ii) satellite-like gold nanocages (AuNCs) with precisely controlled surface density on the MNC, delivering concurrent PTT and PDT. Crucially, anti-epithelial cell adhesion molecule (anti-EpCAM) conjugation substantially augments the binding affinity of TCSN to EpCAM-expressing CTCs, resulting in 3.7-fold greater targeting specificity than that of its nonfunctionalized counterparts (in vitro validation). Microfluidic evaluations using 4T1 cells demonstrated the dual capability of TCSN: dynamic-flow magnetic isolation (92% capture efficiency) followed by photoinactivation (85% viability reduction at 808 nm/0.8 W cm⁻^2^) [161].

### Suppression of NETs formation

**Fig. 4:**
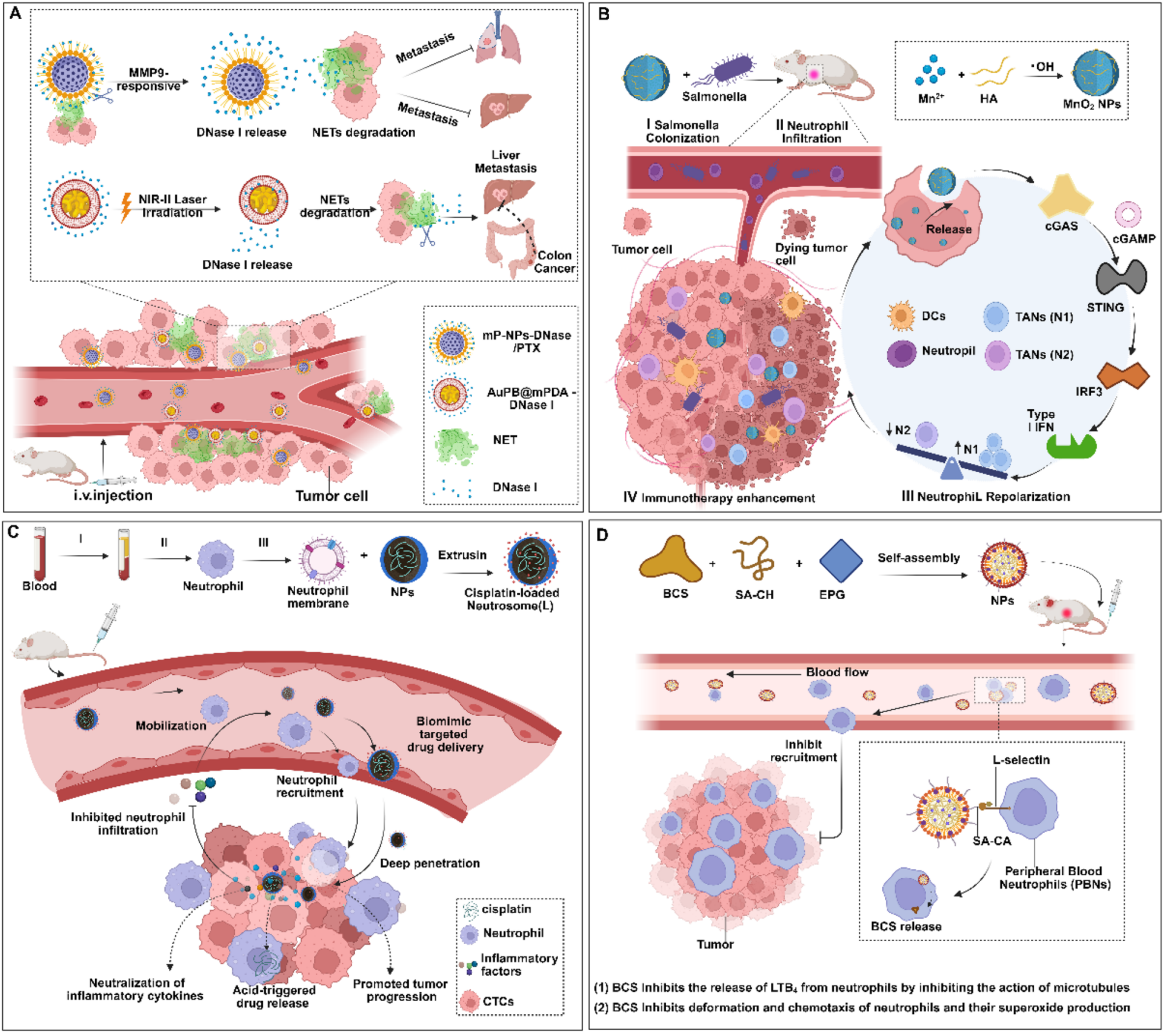
Nanomaterials for driving neutrophil repolarization toward the N1 phenotype, degrading ENTs and modulating neutrophil infiltration. **A** NETs degradation inhibits tumor metastasis. mP-DNase/PTX releases DNase I in the tumor microenvironment to degrade NETs, inhibiting hepatic and pulmonary metastases. AuPB@mPDA-DNase I (AMD) uses NIR-II laser irradiation to control DNase I release, suppressing colorectal cancer liver metastasis through photothermal therapy and NETs degradation. **B** Salmonella establishes intratumoral colonization and promotes neutrophil infiltration into the tumor microenvironment. Subsequently, manganese oxide nanoparticles release Mn^2^⁺ ions, activating the STING pathway to induce type I interferon expression (e.g., IFN-β). This signaling cascade reprograms TANs from protumorigenic N2 phenotypes to antitumor N1 phenotypes. **C**, **D** Nanotherapeutics influence neutrophils recruitment into the tumor microenvironment. **C** Neutrosomes (L) adsorbs tumor-secreted cytokines, reducing neutrophil migration to tumor sites. **D** BC nanotherapeutics bind to L-selectin on neutrophils, inhibiting LTB4 release and neutrophil functions and decreasing neutrophil recruitment to tumors. Created in BioRender. Su, Y. (2025). https://BioRender.com/h54e244 (accessed on 23 March 2025)

NETs sequester circulating tumor cells and promote metastasis [162]. To counteract NET-mediated immune evasion and facilitate the metastatic ability of CTCs, emerging nanotherapeutic strategies focus on NETs inhibition or degradation. Park et al. pioneered this approach via the use of DNase I-coated nanoparticles that enzymatically dismantle the DNA backbone of NETs, liberating trapped CTCs and reducing pulmonary metastases by 62% in murine breast cancer models. Furthermore, DNase I suppressed breast cancer cell migration in vitro by disrupting metastasis-promoting NETs architectures [163]. In addition to this paradigm, Chen et al. developed a photoresponsive nanoplatform (Au-PB@mPDA) comprising a plasmonic gold blackbody core and a mesoporous polydopamine shell for spatiotemporal NETs regulation. NIR-II-triggered DNase I release from the Au-PB@mPDA resulted in localized NETs degradation in the tumor microenvironment, enhancing anti-PD-1 efficacy (tumor growth inhibition increased from 38 to 71%) and suppressing colorectal cancer liver metastasis by 65% [79]. Concurrently, Yin et al. engineered a dual-action nanosystem (mP-NP-DNase/PTX) integrating MMP-9-responsive DNase I delivery with glutathione-triggered paclitaxel release. This smart construct demonstrated (1) 84% NETs degradation efficiency via substrate peptide cleavage and DNase I activation; (2) 3.2-fold greater tumor cell internalization through Tat peptide mediation; and (3) synergistic suppression of primary tumor growth (58% reduction) and distant metastases (73% inhibition) in triple-negative breast cancer models [164] (Fig. 4A). Notably, PAD4 is overexpressed in many cancers, is highly correlated with tumor growth and metastasis, and has an important function in NETs formation [165]. Zhu et al. developed a self-assembled PAD4 inhibitor (ZD-E-1) that exploits dual targeting mechanisms to suppress tumor progression. By integrating PH-responsive binding to PAD4 and tumor-specific accumulation via the enhanced permeability and retention (EPR) effect, ZD-E-1 achieves selective antitumor activity. The inhibitor blocks NETs formation and concurrently enhances immunogenicity by remodeling the tumor microenvironment. These dual actions result in significant inhibition of tumor growth and metastasis, suggesting a promising strategy for precision cancer therapy [166]**.**

NE, a critical enzyme in NETs formation, facilitates chromatin decondensation by histone degradation, and its inhibition effectively blocks NETosis [165]. For atherosclerosis therapy, Shi et al. engineered a liposomal nanoparticle system modified with cyclic arginine-glycine-aspartic acid (cRGD) to target the plaque neovasculature and coloaded the elastase inhibitor livestat (SVT), creating a plaque-targeted "neutrophil hitchhiking" delivery strategy. This approach significantly reduces NETs formation by suppressing intraplaque NE activity, thereby stabilizing atherosclerotic lesions [167]. Furthermore, Hill et al. addressed NE hyperactivation in respiratory diseases (e.g., chronic obstructive pulmonary disease, cystic fibrosis) by conjugating secretory leukocyte protease inhibitor (SLPI) to alginate/chitosan nanoparticles, enabling targeted NE inhibition in pulmonary inflammatory regions [168]. Moreover, Okeke et al. engineered a neutrophil elastase inhibitor delivery system by encapsulating sodium (Sive) in interbilayer-crosslinked multilamellar vesicles (ICMVs), which formed ICMV-Sive nanoparticles. This platform enables rapid neutrophil internalization and effectively suppresses NETs formation in vitro. In vivo studies have demonstrated that ICMV-Sive significantly inhibits NETosis in an endotoxin shock model while prolonging animal survival [169]. While these strategies do not directly investigate NETs interactions with CTCs, they provide critical insights into the inhibition of NE activity to mitigate NET-mediated CTCs metastasis, underscoring the clinical potential of nanotechnology in regulating the NET‒CTC axis.

The ROS-dependent pathways involved in NETs formation have been extensively characterized [48]. Notably, gold nanoparticles (AuNPs) have demonstrated efficacy in suppressing ROS and reactive nitrogen species (RNS) production in macrophages under hyperglycemic conditions, suggesting a potential strategy to mitigate oxidative stress-driven NETs formation [170].

### Inducing the polarization of neutrophils in the tumor microenvironment from the N2 phenotype to the N1 phenotype

Tumor-associated neutrophils (TANs) exhibit phenotypic plasticity, manifesting as suppressive (N1) or tumor-promoting (N2) subpopulations under the regulation of TME signaling networks [61]. A therapeutic strategy targeting this plasticity involves reprogramming protumorigenic N2 TANs into antitumor N1 phenotypes to amplify immune-mediated tumor suppression. Lu et al. exemplified this approach by engineering a synergistic platform combining Salmonella (Sal) and manganese dioxide nanoparticles (MnO₂ NPs). The system operates through a sequential mechanism: (1) Sal establishes intratumoral colonization to recruit neutrophils into the TME; (2) MnO₂ NPs degrade in the hypoxic TME, releasing Mn^2^⁺ ions that activate the STING pathway to induce type I interferon (e.g., IFN-β) expression; and (3) IFN-β subsequently reprograms neutrophils from the N2 phenotype to the N1 phenotype, reshaping the immunosuppressive TME and enhancing the oncolytic activity of Sal. This paradigm underscores the potential of phenotypic reprogramming to synergize bacterial therapy with immunomodulatory nanomaterials for cancer treatment [171] (Fig. 4B).

### Suppression of neutrophil infiltration in the tumor microenvironment

Neutrophils, pivotal inflammatory regulators within the tumor microenvironment, critically influence cancer proliferation, metastasis, and immunosuppressive modulation [172]. Current therapeutic strategies focus on disrupting neutrophil infiltration by targeting chemotaxis or adhesion mechanisms. Neutrophil membrane-coated nanoparticles (NM-NPs) exemplify this approach by leveraging inherited cytokine receptors to intercept inflammatory mediators and chemokines, thereby suppressing the recruitment of both neutrophils and macrophages to inflamed sites [173, 174]. Building upon this foundation, Wu et al. developed a neutrophil-mimetic nanocarrier (Neutrosome (L)) composed of activated neutrophil membrane-cloaked cisplatin-loaded liposomes. Compared with free drug administration, this system capitalizes on the tumor-homing ability of neutrophil membranes to increase cisplatin accumulation in tumor tissues by 4.1-fold. Crucially, Neutrosome (L) adsorbs tumor-derived inflammatory cytokines (e.g., IL-8/CXCL1), disrupting chemotactic gradients and reducing neutrophil migration toward tumor sites by 68%. These combined effects limit neutrophil infiltration while reprogramming the immunosuppressive microenvironment, ultimately amplifying chemotherapeutic efficacy [175, 176]. Additionally, Chen et al. designed a nanodelivery platform targeting peripheral blood neutrophils (PBNs) aimed at reducing the infiltration of tumor-associated neutrophils. This platform is based on a novel low-toxicity colchicine derivative (BCS) nanocomposite material and is surface modified with sialic acid and cholesterol (SA-CH) derivatives. All PBNs express L-selectin on their surfaces, and sialic acid (SA), which acts as a selectin ligand, enables precise targeting [177]. Colchicine, a potent anti-inflammatory drug, inhibits microtubule function to block the release of leukotriene B4 (LTB4) by neutrophils. It also suppresses neutrophil deformation, chemotaxis, and superoxide anion production. Additionally, colchicine binds tubulin in tumor cells, preventing monomer association and polymerization, thereby inducing broad-spectrum antitumor effects [178]. The experimental results demonstrated that the SA-CH-modified BCS formulation is efficiently taken up by neutrophils, significantly inhibiting cell migration, reducing the infiltration of tumor-associated neutrophils, and enhancing T lymphocyte function. In the S180 tumor model, this formulation exhibited excellent tumor-suppressive effects. Notably, in a triple-negative breast cancer model, the drug effectively inhibited tumor metastasis to the lungs [179] (Fig. 4C, D).

### Blockade of Neutrophil–CTC Clusters Assembly

Neutrophil–CTC clusters have been identified as critical facilitators of metastatic progression by shielding CTCs from immune clearance. To disrupt this pathological synergy, three innovative nanotherapeutic strategies have demonstrated targeted intervention mechanisms:

#### Activated neutrophil membrane nanoplatforms (aNEM NPs)

**Fig. 5:**
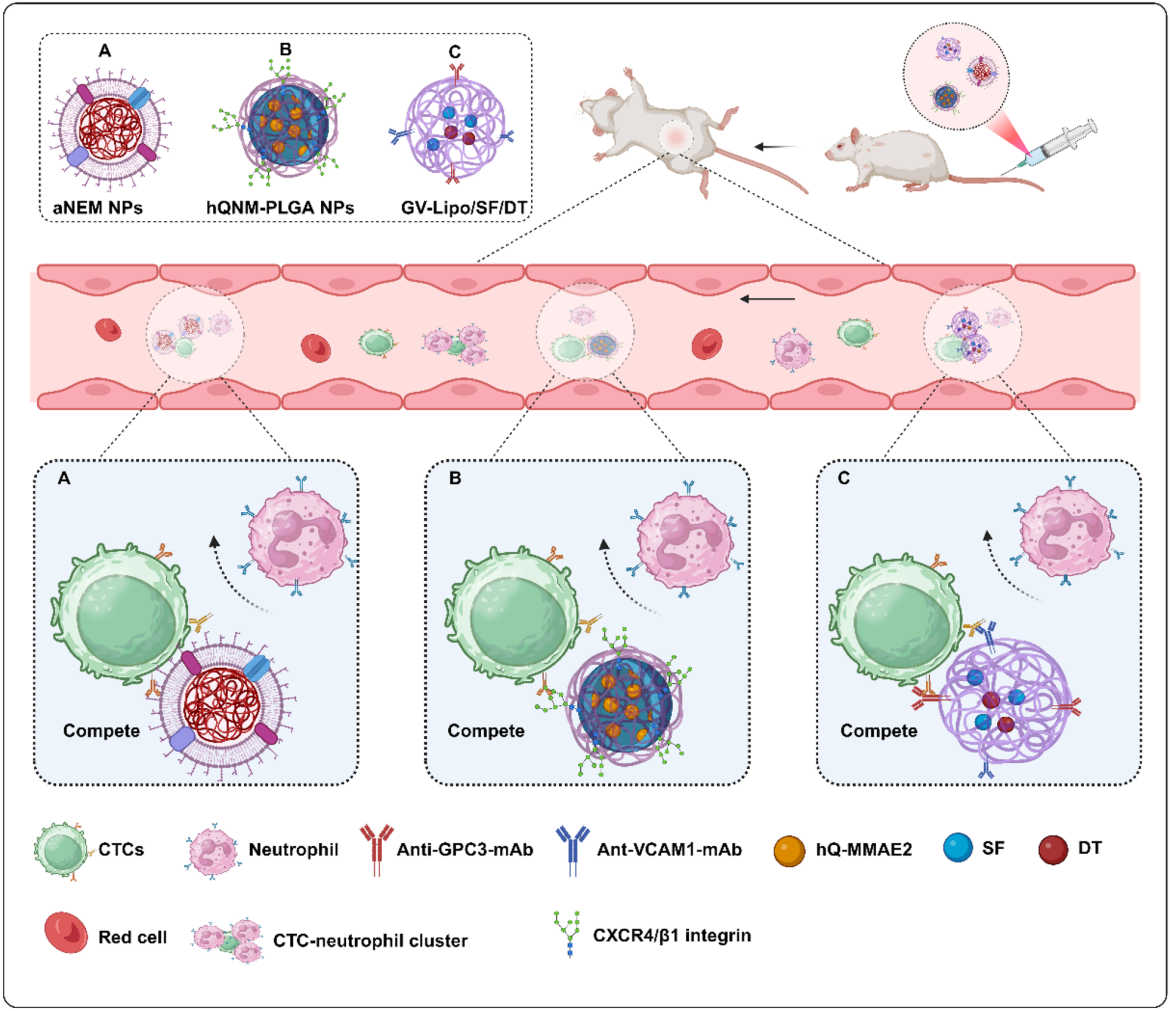
Shows a schematic representation of the disruption of CTC‒neutrophil clusters formation. This process can be achieved by either encapsulating neutrophil membranes or decorating nanoparticles with specific antibodies, such as anti-VCAM1 monoclonal antibodies (mAbs). These approaches enable competitive binding to CTCs, effectively preventing the aggregation of neutrophils with CTCs and thereby inhibiting the formation of CTC‒neutrophil clusters. Created in BioRender. Su, Y. (2025) https://BioRender.com/h54e244 (accessed on 11 March 2025)

Zeng et al. created membrane-coated poly (lactic acid) nanoparticles that competitively inhibited neutrophil recruitment to primary tumors (58% reduction) and premetastatic niches. In vivo studies revealed that aNEM NPs decrease 4T1-derived multiorgan metastases by 65% through disruption of CTC-endothelial adhesion (ICAM-1 binding inhibition = 82%) and cluster formation. These systems collectively establish neutrophil–CTC clusters disruption as a transformative paradigm in metastatic intervention, synergizing immunomodulation with precision chemotherapy through biomimetic nanotechnology [180] (Fig. 5A).

#### Hypoxia-responsive neutrophil-mimetic system (hQNM-PLGA)

Hung et al. engineered PLGA nanoparticles cloaked with neutrophil membranes and loaded them with a hypoxia-activated MMAE2 prodrug (hQ-MMAE2). This biohybrid system achieves dual functionality: (a) competitive inhibition of neutrophil-CTC clusters formation through membrane-derived adhesion molecules, reducing CTCs extravasation by 68% in advanced 4T1 mammary tumors; (b) hypoxia-triggered MMAE2 release (tumor-to-normal tissue ratio = 5.3:1), resulting in 74% primary tumor suppression [181] (Fig. 5B).

#### Multi-target HCC nanodevice (GV-Lipo/SF/DT)

Mu et al. developed an innovative multitarget combinatorial therapy platform, GV-Lipo/SF/DT, designed to simultaneously target primary tumors and associated CTCs for enhanced metastasis suppression and therapeutic efficacy against hepatocellular carcinoma (HCC). This advanced nanotherapeutic system integrates sorafenib (SF) and digitoxin (DT) into a single liposomal carrier, with the surface of the coloaded liposomes further functionalized with anti-GPC3 monoclonal antibodies (mAbs) and anti-VCAM1 mAbs [182]. DT, an FDA-approved potent Na^+^/K^+^-ATPase inhibitor, has the exceptional ability to dissociate CTC clusters [183], whereas SF, the first FDA-approved multikinase inhibitor for first-line treatment of HCC, effectively eliminates primary tumor cells and prevents metastasis through specific targeting [184]. Experimental studies in H22 tumor-bearing models and orthotopic HCC models revealed that this nanosystem leverages enhanced glypican-3 (GPC3) and tumor vascular cell adhesion molecule-1 (VCAM1) targeting to recognize and capture CTCs. GPC3, a cancer/testis-associated glycoprotein highly expressed in HCC, serves as a key receptor for targeted recognition [185]. The system operates through a sequential mechanism: DT dissociates CTC clusters, SF eradicates individual CTCs, and importantly, anti-VCAM1 mAbs block the formation of CTC‒neutrophil clusters by binding to overexpressed VCAM1 on CTCs membranes. Furthermore, the dual-targeting strategy utilizing anti-VCAM1 mAbs and anti-GPC3 mAbs enhances the specificity and sensitivity of therapy toward both CTCs and primary tumor cells, thereby improving overall therapeutic outcomes [41, 163] (Fig. 5C).

These studies highlight the potential of nanomedicine for targeting CTCs, NETs, and CTC–neutrophil clusters. By exploiting the unique characteristics of these cells, nanomedicine facilitates the selective delivery of therapeutic agents, thereby modulating their functions and interactions. This innovative approach could pave the way for novel strategies for the treatment of cancer metastasis.

## Nanomaterial-based drugs that target CTC-neutrophil clusters: perspectives and challenges

Research on nanomaterial-based drugs targeting CTC‒neutrophil clusters is a rapidly evolving field. The development of this field not only offers more precise and effective treatment options for patients with metastatic cancer but also holds promise for elucidating the mechanisms by which neutrophils contribute to cancer metastasis. However, translating this innovation into clinical practice presents numerous challenges and limitations that must be addressed. The following sections explore future perspectives and challenges in this field from multiple angles.

### Complexity of the immune environment in biological systems

Targeting CTCs and NETs, reprogramming of neutrophil phenotypes and modulating CTC‒neutrophil interactions represent promising strategies for preventing or inhibiting cancer metastasis. However, the complexity of the immune environment within biological systems poses significant challenges. Upon entering the human body, nanomaterials may elicit immune responses, leading to their rapid clearance. Additionally, nanomaterials may nonspecifically bind to other cells or tissues, potentially interfering with the normal functions of unrelated immune cells. This can reduce targeting efficiency and compromise therapeutic outcomes. To address these challenges, integrating surface modifications of nanoparticles, such as polyethylene glycosylation (PEGylation), with biomimetic designs, such as those that mimic the molecular structure of cellular membranes, can enhance biocompatibility and minimize immunogenicity. Concurrently, the development of nanocarriers with enhanced precision in terms of pH responsiveness, enzyme responsiveness, and photoresponsiveness is critical. Furthermore, by optimizing the physical and chemical properties of nanomaterials, including their size, shape, and surface charge, sensitive responses to external stimuli, precise drug release, and reduced interactions with nontarget cells can be achieved, and the membrane coating efficiency for cell membrane-mimicking nanosystems can be optimized [138, 186]. These advancements could significantly improve therapeutic efficacy while minimizing off-target effects. In summary, overcoming the challenges associated with nanomaterial-based therapies requires a multifaceted approach that combines advanced material design, precise stimulus-responsive functionality, and a deep understanding of the immune microenvironment. These innovations hold great potential for advancing cancer therapy and improving patient outcomes.

### Heterogeneity of CTC‒neutrophil clusters

The design of nanomaterials with high specificity for the precise targeting of specific types of CTC‒neutrophil clusters represents a critical challenge in the field. However, the heterogeneity of CTC‒neutrophil clusters, coupled with their interactions with the tumor microenvironment or other cells and factors in the circulation, poses significant obstacles to achieving selective targeting. To address these challenges, it is essential to develop "multifunctional nanoplatforms" that integrate multiple targeting ligands, such as antibodies, peptides, or small molecules, to enable multimodal recognition and precise targeting of CTC‒neutrophil clusters. This approach not only enhances the specificity of targeting but also provides a robust platform for overcoming the complexity and variability inherent in these biological systems.

### Potential toxicity and long-term safety concerns of nanomaterials

Analyzing the distribution and metabolism of nanomaterial-based therapeutics within the body is crucial for understanding their biological characteristics and clinical significance. However, owing to the limitations of current in vivo tracking technologies for nanomaterials, the precise metabolic pathways and clearance mechanisms of these materials remain incompletely understood. This lack of clarity could lead to toxic effects on normal cells or organs, particularly during long-term use. Therefore, it is imperative to integrate advanced in vivo imaging techniques with metabolomics data to thoroughly investigate the dynamic behavior of nanomaterials in vivo. Additionally, the development of biodegradable nanomaterials could enhance metabolic controllability, offering a promising strategy to address these challenges comprehensively.

Several nanotherapeutic systems have been clinically approved for the treatment of metastatic cancer, including irinotecan liposomes for gemcitabine-refractory metastatic pancreatic cancer, DOX liposomes for metastatic breast cancer, cytarabine liposomes for leptomeningeal metastases, and vincristine sulfate liposomes for metastatic melanoma [97]. These advancements provide critical insights into the mechanisms by which CTC–neutrophil cluster–targeted nanosystems inhibit cancer metastasis.

## Conclusion

In summary, tumor metastasis has garnered significant attention because of its dismal survival rates and suboptimal therapeutic outcomes. Conventional therapeutic modalities, including surgery, chemotherapy, and radiotherapy, have demonstrated limited efficacy in addressing this challenge, with accumulating evidence suggesting that these interventions (particularly surgical resection and chemotherapeutic agents) may paradoxically potentiate metastatic dissemination. Despite the formidable obstacles in antimetastatic therapy, nanotechnology-driven drug delivery systems (DDSs) have achieved promising breakthroughs. Leveraging their unique physicochemical properties, nanoscale particulates not only optimize drug-loading capacities but also substantially mitigate off-target toxicity through reduced exposure of healthy tissues to therapeutic agents. This review systematically examines nanoplatforms engineered to suppress CTC‒neutrophil clusters formation, thereby preventing, attenuating, or eradicating neutrophil-facilitated metastatic potential.

As delineated in this review, neutrophil-mediated tumor metastasis involves multiple biological processes, including the induction of tumor cell genomic instability, cytokine secretion, T-cell activity suppression, tumor angiogenesis, extracellular matrix remodeling, interactions with CTCs, and NETs formation. The strategic application of nanocarriers loaded with antitumor therapeutics (e.g., radiotherapy agents, chemotherapeutic drugs, immunotherapies, or combination regimens), coupled with bioinspired cell-based strategies, leverages NP surface modifications to achieve active targeting of CTCs and NETs, thereby providing a promising strategy for effective and safe suppression of neutrophil-facilitated metastasis. Nevertheless, critical challenges persist in bioinspired nanosystems, particularly immunogenicity concerns, unpredictable biological responses, nonstandardized manufacturing protocols, suboptimal cell membrane extraction techniques, and long-term storage instability—limitations that significantly impede their clinical translation.

Given the transformative potential of such nanosystems in metastatic cancer therapy, future investigations should prioritize overcoming these technical bottlenecks, including immunogenicity modulation, standardization of biofabrication protocols, and optimization of long-term stability profiles, to facilitate their scalable clinical translation. This necessitates interdisciplinary collaboration integrating advanced nanotechnology, immunopharmacology, and good manufacturing practice (GMP)-compliant production methodologies (Table 2).

**Table 2:** Nanomedicine reducing CTC-neutrophil clusters formation

Mechanism of action	Cell membrane	Nanoparticle delivery platform	Drug	Disease model	Cell line	References
Direct therapeutic targeting of CTCs	Neutrophil	NM-NP-CFZ	CFZ	Lung metastatic mouse model	4T1	[109]
	Cancer cell-derived exosomal	EMPCs	PTX-S-LA CuB	Orthotopic and xenograft MDA-MB-231 tumor models	MDA-MB-231	[114]
	Platelet	PMDIs	DOX ICG	Breast cancer metastasis model	MDA-MB-231	[118]
		TRAIL-Dox-PM-NV	TRAIL DOX	Breast cancer metastasis model	MDA-MB-231	[119]
		TRAIL@PMDV-SiO_2_ NPs	TRAIL	Breast cancer metastasis model	MDA-MB-231, PC3	[122]
		aPD-L1-PM-SO@HMSNs	SO aPD-L1	HCC	H22	[124]
	Macrophage	Dox-MPK@MDL	DOX	BCLM	4T1	[126]
	Hybrid	hNVs	–	TNBC/MM	4T1	[127]
		PNMAuDIs	DOX ICG	4T1 back tumor-bearing mice models	4T1	[133]
		Fe_3_O_4_-ICG@HM	ICG	–	MCF-7 cells	[136]
	–	Es/TRAIL-loaded liposome	TRAIL	Orthotopic PC xenograph model	–	[147]
		Es/TRAIL-loaded liposome	TRAIL	PC	DU145 cells	[148]
		Es/TRAIL-Loaded Liposome	TRAIL	Colon cancer pulmonary metastasis model	COLO 205, PC-3	[149]
		ES-PEG L-DXR	DOX	–	COLO 205, MCF7	[150]
		dTNP	PTX	4T1-GFP-LM	4T1-GFP	[152]
		Rg3-Lp/DTX	DTX	TNBC	4T1	[197]
		PM-NCS:Fab	NCS	CRC	HCT116, HCT8 and HT29	[155]
		PM-Zileuton™	Zileuton™	TNBC	MCF-7 and MDA-MB-231	[157]
		uPtDs NPs	Pt(II)	TNBC	MDA-MB-231, LM2 and SUM159	[159]
		TCSN	PTT PDT	–	4T1	[161]
Suppression of NETs formation	–	DNase I NPs	DNase I	Breast cancer model	4T1, 4T07 cells	[163]
	–	Au-PB@mPDA	DNase I	CRC	–	[79]
	–	mP-NPs-DNase/PTX	DNase I PTX	Breast cancer model	A549 cells, and 4T1	[164]
	–	ZD-E-1	ZD-E-1	–	4T1, LLC and S180 cells	[166]
	–	cRGD-SVT-Lipo	Sivelestat	Atherosclerosis	–	[167]
	–	SLPI-loaded alginate/chitosan particles	SLPI	CF	–	[168]
	–	ICMV-Sive	sivelestat	–	–	[169]
	–	AuNPs	–	Atherosclerosis	RAW264.7, and THP-1	[170]
Reprogramming of neutrophil phenotypes	–	MnO₂ NPs	*Salmonella* and MnO2	4T1 tumor-bearing BALB/c mice	4T1	[171]
Suppression of neutrophil infiltration in tumor microenvironment	Neutrophil	NM-NPs	Pt(II)	A549 subcutaneous transplantation tumor model	A549 cells	[175]
	–	SA-EPG-BCS	BCs	4T-1 breast cancer model	S180 murine sarcoma cell lines and 4T-1 cells	[179]
					–	
			DNase I		A549 cells, and 4T1 cells	
Blockade of neutrophil- CTC clusters assembly	Neutrophil	hQNM-PLGA	hQ-MMAE2	Breast cancer model	4T1	[181]
	–	GV-Lipo/SF/DT	SF DT	H22‐bearing tumor model and orthotopic HCC models	H22 cells, and Hepa1‐6 cells	[182]
	Neutrophil	*aNEM NPs*	–	Breast cancer model	4T1	[180]

CFZ, carfilzomib; CuB, cucurbitacin B; PTX-S-LA, paclitaxel-linoleic acid prodrug; DOX, doxorubicin; NCS, nicorandil; Pt(II), cisplatin; CLC, colchicine; DT, digitoxin; TRAIL, TNF-related apoptosis-inducing ligand; aPD-L1, anti-PD-L1 antibody; DNase I, deoxyribonuclease I; PTT, photothermal therapy; PDT, photodynamic therapy; ICG, indocyanine green; hQ-MMAE2, hydroquinone-monomethyl auristatin E conjugate; HCC, hepatic cell carcinoma; BCLM, lung metastasis of breast cancer; TNBC, triple-negative breast cancer; MM; B16F10 mouse melanoma model; PC, prostate cancer; 4T1-GFP-LM, 4T1-GFP cell-derived lung metastasis mice model; CRC, colorectal cancer; 4T1, mouse breast carcinoma cells; THP-1, human monocytic cells; RAW264.7, murine macrophage; SLPI, secretory leukocyte protease inhibitor; CF, cystic fibrosis; S180, mouse sarcoma S180 cells; LLC, mouse Lewis lung cancer cells

## Data Availability

No datasets were generated or analysed during the current study.
